# CHILDREN WITH MULTIPLE CONGENITAL DEFECTS: WHAT ARE THE LIMITS BETWEEN THERAPEUTIC OBSTINACY AND THE TREATMENT OF UNCERTAIN BENEFIT?

**DOI:** 10.1590/1984-0462/;2017;35;1;00004

**Published:** 2017-02-20

**Authors:** Patricia Souza Valle Cardoso Pastura, Marcelo Gerardin Poirot Land

**Affiliations:** a Fundação Oswaldo Cruz (FIOCRUZ), Rio de Janeiro, RJ, Brasil.; b Universidade Federal do Rio de Janeiro (UFRJ), Rio de Janeiro, RJ, Brasil.

**Keywords:** pediatrics, ethics, congenital abnormalities

## Abstract

**Objective:**

Therapeutic approach of children with multiple malformations poses many dilemmas, making it difficult to build a line between the treatment of uncertain benefit and therapeutic obstinacy. The aim of this paper was to highlight possible sources of uncertainty in the decision-making process, for this group of children.

**Case description:**

An 11-month-old boy, born with multiple birth defects and abandoned by his parents, has never been discharged home. He has complex congenital heart disease, main left bronchus stenosis and imperforate anus. He is under technological support and has gone through many surgical procedures. The complete correction of the cardiac defect seems unlikely, and every attempt to wean the ventilator has failed.

**Comments:**

The first two main sources of uncertainty in the management of children with multiple birth defects are related to an uncertain prognosis. There is a lack of empirical data, due to the multiple possibilities of anatomic or functional organ involvement, with few similar cases described. Prognosis is also unpredictable for neuro-developmental evolution, as well as the capacity for the development and regeneration of other organs. Another source of uncertainty is how to qualify the present and future life as worth living, by weighing the costs and benefits. The fourth source of uncertainty is who has the decision: physicians or parents? Finally, if a treatment is defined futile then, how to limit support? No single framework exists to help these delicate decision-making processes. We propose, then, that physicians should be committed to develop their own perception skills in order to understand patient’s manifestations of needs and family values.

## RESUMO

**Objetivo:**

A abordagem terapêutica de crianças com múltiplas malformações inclui muitos dilemas, tornando difícil diferenciar um tratamento de benefício duvidoso da obstinação terapêutica. O objetivo deste artigo foi destacar as possíveis fontes de incerteza no processo de tomada de decisão para esse grupo de crianças.

**Descrição do caso:**

Lactente de 11 meses de idade, que nasceu com múltiplas malformações congênitas e foi abandonado por seus pais, nunca recebeu alta hospitalar. Ele tem cardiopatia congênita cianótica, estenose do brônquio fonte esquerdo e imperfuração anal. Passou por muitos procedimentos cirúrgicos e permanece sob suporte tecnológico. A correção total do defeito cardíaco parece improvável, e todas as tentativas de desmame do ventilador falharam.

**Comentários:**

As duas principais fontes de incerteza no processo de tomada de decisão para crianças com múltiplos defeitos congênitos estão relacionadas ao prognóstico incerto. Dados empíricos escassos são por conta das múltiplas possibilidades de envolvimento (anatômico ou funcional) de órgãos, com poucos casos semelhantes descritos na literatura. O prognóstico é também imprevisível para a evolução da capacidade cognitiva e para o desenvolvimento de outros órgãos. Outra fonte de incertezas é como qualificar uma vida como valendo a pena ser vivida, ponderando custos e benefícios. A quarta fonte de incerteza é quem tem a decisão: os médicos ou os pais? Finalmente, se um tratamento é definido como fútil, então, como limitar o suporte? Na ausência de um método universal para essa tomada de decisão, ficamos com a responsabilidade dos médicos em desenvolver suas habilidades de percepção das necessidades dos pacientes e dos valores familiares.

## INTRODUCTION

Birth defects and congenital diseases are the leading cause of neonatal mortality in the United States,^1^ and they already represent the second leading cause of mortality in the first year of life in Brazil.^2^ Despite the overall frequency, each case with a specific association of defects tends to be unique and helps to determine an unknown prognosis. There is no framework to make decisions over treatment and support for these children. 

Our main objective in reporting this case was to highlight the conflicts and uncertainties that arise in the treatment of children with multiple malformations. We are especially concerned about the limits of therapeutic approaches of uncertain benefit with others characterized as therapeutic obstinacy.

## CASE DESCRIPTION

An 11-month-old boy born with multiple congenital malformations has never been discharged home. He spent his first three months in neonatal intensive care unit, where he has gone through some interventions, such as Blalock-Taussig procedure for complex cyanotic heart disease, tracheostomy for congenital tracheal stenosis, and gastrostomy to lower bronchoaspiration risk. Since his birth, he required ventilator support and, despite many attempts, he was never weaned. He underwent chest tomography and bronchoscopy, which also diagnosed the left main bronchus stenosis. Besides the heart and airways double lesion, he has other malformations: grade V unilateral vesicoureteral reflux and anal imperforation. He is on antimicrobial prophylaxis and he has a colostomy.

Daily procedures such as tracheostomy aspirations cause much suffering, which is manifested by intense sweating, severe cyanosis, and sometimes the need for urgent cannula replacement. Several arterial punctures were performed for blood analysis and vein punctures for peripheral or central venous accesses. In addition, he had to be treated twice for bronchial hyperreactivity with intravenous beta-2 agonist and ventilation under muscle paralysis. Another life-threatening event was a hypovolemic shock secondary to severe bleeding following an airway dilatation procedure. There were two more episodes of dilation by bronchoscopy and a cardiac catheterization, totalizing six anesthetic procedures.

Meanwhile, his ability to interact socially leaves the health care team amazed. Despite an expected developmental delay due to prolonged hospitalization, he is achieving milestones: clapping hands and sending kisses.

We do not know how much discussion was held regarding the limits of treatment before the performance of each procedure, such as tracheostomy and the first cardiac surgery. Currently, the problematic issue is whether to submit him to definitive cardiac procedure or initiate palliative care. The heart surgery involves technical difficulties for total correction, and it probably will not make ventilator weaning easier. On the other hand, palliative care holds many possibilities and they have never been discussed. Withdrawing life support was never an issue.

The decisions became more difficult in this case because they rely totally on the medical staff. The family has poor emotional bond with the child. Despite the possibility of aid provided by local social service, for transport fees, for example, parents rarely visit him or make phone calls to enquire about the child.

## COMMENTS

### Definitions and uncertainties

A possible definition of therapeutic obstinacy or medical futility is the treatment and support that cannot cure the patient, but merely prolong his life in harsh conditions. The American Society of Critical Care Medicine classifies futile treatment as the one that serves no purpose and has no beneficial physiologic effect. Other categories include treatments that are judged inappropriate and hence inadvisable, but not futile. They are:

1. treatments extremely unlikely to be beneficial;
2. beneficial treatments extremely costly;
3. treatments of uncertain benefit.^3^

In the specific case of children with multiple congenital anomalies, there are three major sources of uncertainties regarding this boundary between treatment of dubious benefit and therapeutic obstinacy. First, there is the ill-defined prognosis, which is determined by the rare occurrence of anatomic and physiologic defects. Prognosis is not well described even for diseases that are more common. The second source of uncertainty is related to the unpredictability of the burden of the disease in a child’s growing and developing organism. Finally, there are uncertainties derived from the attempt to qualify each singular life as a life worth living, by weighing suffering and joy.

### Uncertainties about prognosis

The word *prognosis*, meaning a prediction, already holds the dimension of uncertainty. The outcome is generally difficult to predict due to individual variability, differences in progression, and stages of diseases and the possibility of comorbid conditions. Each patient is always unique.

Anyway, when a disease is frequent enough, probabilist data are derived from series and samples. It is even possible to produce guidelines that support decision-making. An example is prematurity and the problematic question in delivery room: to resuscitate or not. Many infants evolve to chronicity, technology dependence, and high risk of handicap. This subject is much explored in the literature^4^ and there are plenty data available to establish the “viability limit”, i.e., gestational age at which there is a reasonable chance of long-term survival, determining full intervention.

Infants with birth defects, born prematurely or not, pose an even bigger dilemma. There are countless possibilities of single malformations, associations, or syndromes - almost all rare in occurrence. This scenario of prognosis uncertainty is determined mostly by lack of empirical data.

The other source of uncertainty over prognosis is not related to the course of a specific disease. What is unknown is childhood capacity for organ development and regeneration. A classical example is the anatomic and functional plasticity of immature and not yet fully developed young child’s brain. Cognitive capacity is difficult to predict in cases of injury or malformation.^5^ Respiratory function is another example: it is known to improve with age in children who are chronically ventilated by pulmonary, cardiac, or muscular diseases.^6^

Finally, even when it is possible to determine survival rates and the probabilities of long-term morbidities, these chances are not the same thing as to define what is good or acceptable.^7^

### Uncertainties in characterizing a life as worth living

A general approach to uncertainties about treating or not children with multiple birth defects is to measure the quality of present and future life, by weighting costs and benefits.^8^ However, there seems to be no universal rules to be applied in this process of evaluating lives that are worth living. So, many doubts are raised for each situation.

Catlin, for example, suggests some issues to be addressed for children with trisomy 18.^9^ Should we really support an approach of technological interventions and multiple surgical corrections - heart, palate, limbs, esophagus - organ by organ? How many surgeries will that be? Will the child suffer through them, especially if there are multiple and sequential procedures? Should treatment scale up each organ dysfunction? Should we maintain full-intervention approach even if cognitive capacity is knowingly to be compromised? Is it desirable that a disabled child survives their parents’ death and caregivers in the future? Can decisions be reviewed?^9^

Another list of issues to be considered when evaluating quality of life was set by the English Nuffield Council on Bioethics. It addresses benefits for the future life: the likelihood of pleasurable experiences and the establishment of emotional relationships. Other concerns are the child’s ability to live without technological support and outside the hospital.^10^

One must think about all those issues to come to an ethical judgment, but much uncertainty is still left behind. There are uncertainties even about the questions posed. What is acceptable in terms of cognitive and physical impairments? How to value pleasurable experiences? What exactly is the child’s best interests?

Another approach to measure quality of life is the use of the score systems. It is almost a mathematical way to determine whether joys and interaction with family members outweigh physical pain and psychological suffering.^11^ In fact, this methodology transforms qualitative into quantitative analysis, building a limit between pleasures and pain. Above this limit, the neutrality threshold, there is a life that is worth living.^12^

Determining the limits of a life that is worth living, however, seems somehow reductionist. A critic is that biomedical scrutiny of objective facts, such as a sum of disabilities, impairment, and survival probabilities, is prioritized over subjective experiences.^11^ In fact, decision-makers should not expect general and strict rules of action to be appropriate to every singular situation. They need to try and understand patients’ perspectives and experiences. To judge the quality of a chronically ventilated child life, for example, it is necessary to determine how much discomfort aspirations cause.^13^ Paediatricians also need to increase their abilities to recognize the efforts that some children manifest in a struggle for survival.^10^

### Fourth source of uncertainty: decision-makers

Some authors consider that parents and family members should have autonomy or the authority by proxy over decisions in the best interests of their children. These decisions occur in socio-cultural and family values contexts.^14^ ^,^ ^15^ Physicians should only acknowledge each family’s reality and avoid strict rationalism in technical decisions.^16^ On the other hand, European and Latin-American doctors usually think of themselves as bearing the major burden of decision-making.^17^ They also think parents have impaired judgments in stress situations and should be protected from guilty over the irreversible decisions.^18^

Currently, shared decisions are the consensus supported by the American Academy of Pediatrics.^19^ ^,^ ^20^ Shared decision-making, however, is not easily practiced, and occurs within a *continuum* of possibilities. It varies from decisions that depend mostly on physicians to decisions that are mostly parents-driven. The final goal should be a consistent decision with patients and parents’ wishes, beliefs, and values.

To understand patient’s perspectives and values, physicians need to recognize relational deficiencies and develop some perception skills and the ability to listen. This may represent a cultural change. The qualities they should possess include compassion, humility, and courage, in as much as the capacity to be prepared in order to live with their own doubts.^13^

Therapeutic futility and limitation of life support

As presented so far, uncertainties surround the management of children with multiple birth defects. However, if the treatment is already defined as futile, the decision should be against the maintenance of life at any cost. Obstinacy may only reflect the inability to accept the limits of treatment and death, based on the beliefs of reverence for life or in the curative powers of medicine.

In the 1990s, Dunn already considered justifiable life support limitation for three groups of infants: extremely premature with serious problems like periventricular hemorrhage, those with severe malformations and the ones with severe neurological injury.^21^

Once a decision was made for life support limitations, new uncertainties arise regarding legal and biomedical issues. Legal aspects can be exemplified by the existence or not of a so specific end-of-life legislation to be followed, or a local Bioethics Committee for consultation. The most important biomedical issue to be addressed is the method for life support limitation: withdrawal or withhold therapy and procedures. Currently, the majority of deaths in the intensive care units are due to life support limitation - by means of withholding treatment as a “do not resuscitate” command.^22^ ^,^ ^23^ Extreme premature babies encompass the greatest population. The French Society of Neonatology also considers justified the withdrawal of life-sustaining treatment if the intention is to stop an unreasonable opposition to the natural course of a disease.^24^

When children go through many complications during prolonged hospitalizations, therapeutic support seldom prolongs life, but the dying process.^25^

## CONCLUSIONS

Considering all sources of uncertainty discussed, we assume that there are no simple rules or models to decide whether to invest in therapy and life support for children with multiple birth defects. The boundaries between therapeutic obstinacy and treatment of uncertain benefits remain obscure.

No criterions for precise prognostic determination and no metric method for qualifying a life that is worth living can be used to reach a single answer - the right one. In fact, values are what matter. Formerly, it was about respecting family values. However, not only in this particular abandonment case, as well as in the perspective of shared decision-making, physicians’ values also count. The place a physician occupies in the *continuum* of the decision process is certainly a decision in itself. And it is a responsibility too.

So, we would also like to emphasize on professional responsibility in improving clinical judgment skills for making good choices. It encompasses technical and ethical aspects. Physicians and health care professionals must be able to realize patients’ unlimited expressions of needs and demands.
